# Neural correlates of working memory development in adolescent primates

**DOI:** 10.1038/ncomms13423

**Published:** 2016-11-09

**Authors:** Xin Zhou, Dantong Zhu, Xue-Lian Qi, Sihai Li, Samson G. King, Emilio Salinas, Terrence R. Stanford, Christos Constantinidis

**Affiliations:** 1 Department of Neurobiology & Anatomy, Wake Forest School of Medicine, Winston-Salem, North Carolina 27157, USA; 2 Department of Computer Science, Stanford University, Stanford, California 94305, USA

## Abstract

Working memory ability matures after puberty, in parallel with structural changes in the prefrontal cortex, but little is known about how changes in prefrontal neuronal activity mediate this cognitive improvement in primates. To address this issue, we compare behavioural performance and neurophysiological activity in monkeys as they transitioned from puberty into adulthood. Here we report that monkeys perform working memory tasks reliably during puberty and show modest improvement in adulthood. The adult prefrontal cortex is characterized by increased activity during the delay period of the task but no change in the representation of stimuli. Activity evoked by distracting stimuli also decreases in the adult prefrontal cortex. The increase in delay period activity relative to the baseline activity of prefrontal neurons is the best correlate of maturation and is not merely a consequence of improved performance. Our results reveal neural correlates of the working memory improvement typical of primate adolescence.

## Abstract (web summary)

Working memory is known to improve through adolescence into adulthood, yet the associated changes in neuronal activity are not well understood. Zhou and colleagues report increased delay period activity correlated with changes in performance on working memory tasks in monkeys as they transition into adulthood.

## Graphical abstract

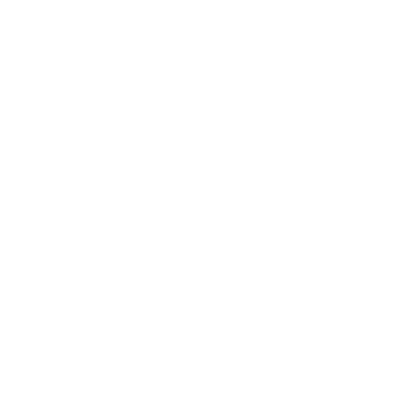

Working memory ability and other cognitive faculties increase considerably between the time of puberty and adulthood^1^,^2^,^3^,^4^,^5^. Human studies indicate a monotonic increase in working memory performance during this time period, particularly for tasks that require retaining information during distraction^1^,^2^,^6^. This prolonged cognitive enhancement that persists after the onset of puberty parallels the maturation of the prefrontal cortex^6^,^7^,^8^,^9^,^10^,^11^. Anatomical changes in the prefrontal cortex that continue throughout adolescence include increases in grey and white matter volumes, and in myelination of axon fibres within the prefrontal cortex and between the prefrontal cortex and other brain areas^6^,^7^,^8^,^9^,^10^,^11^,^12^,^13^. A progressive increase of prefrontal activation between childhood and adulthood has also been well documented via imaging studies for tasks that require working memory and filtering of distractors^12^,^13^,^14^,^15^,^16^,^17^,^18^.

Much less is known about how the physiological properties of prefrontal neurons, which likely mediate these changes, develop during adolescence. Non-human primate models have provided neural correlates of working memory in the persistent activity of prefrontal neurons during the delay period of working memory tasks^19^,^20^. Similar to the human pattern of development, the monkey prefrontal cortex continues to undergo anatomical maturation during adolescence and early adulthood^21^,^22^. By some accounts, however, biochemical and anatomical changes in the prefrontal cortex characteristic of adolescence in humans occur at an earlier, pre-pubertal age in monkeys^23^,^24^, but longitudinal studies in monkeys tracking neural activity changes have been sparse. For these reasons, it is not known if working memory development occurs after puberty in monkeys, or what changes in neural activity mediate cognitive enhancement during development. To address these questions, we tracked developmental markers of monkeys as they transitioned from puberty to adulthood and followed their cognitive development by testing them in working memory tasks. We identified behavioural changes in monkeys around the time of puberty by tracking animals longitudinally and we additionally recorded neural activity from the prefrontal cortex. The results show concomitant changes in the coding capacities of prefrontal neurons during this critical period of development, which mainly involved increased activity during the delay period of the task.

## Results

### Subjects

Four male macaque monkeys (*Macaca mulatta*) were used in this study. Behavioural and neural experiments were performed at two stages of development: after the onset of puberty (which we refer to as the ‘young' stage) and in adulthood (‘adult' stage). The time of puberty was determined based on morphometric, radiographic and hormonal measures (see the ‘Methods' section). The first round of neurophysiological recordings was obtained after the onset of puberty, while the monkeys were on a growth trajectory. The last measurement before the onset of neurophysiological recordings corresponded to a median age of 4.3 years (range: 4.0–5.2 years). This experimental stage lasted 3–6 months. Monkeys were then returned to their colony and received no further training or exposure to any behavioural task for ∼1 year. The monkeys were briefly re-introduced to the task and a second round of recordings was obtained. The median age of animals at the onset of the second stage of experiments was 6.3 years (range of ages: 5.6–7.3; range of intervals from young stage: 1.6–2.1 years). Morphometric measures had plateaued at the time of recordings in the adult stage.

### Behavioural performance

**Figure 1:**
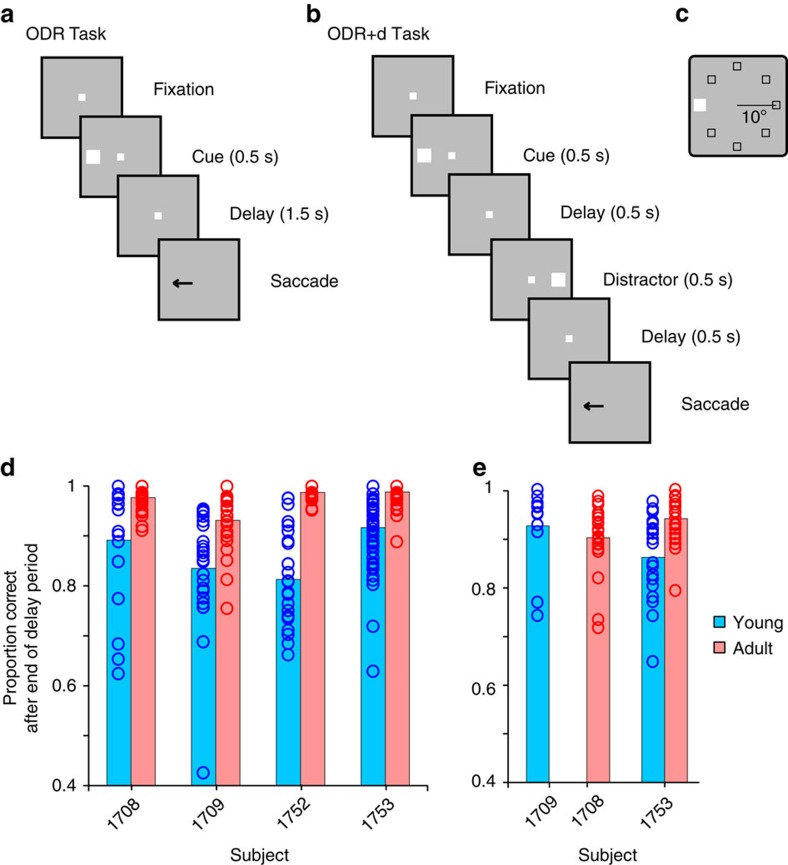
Task and behavioural performance. (**a**) Frames represent sequence of events in the ODR task. The monkey is required to observe a cue stimulus, maintain fixation during a delay period and, when the fixation point turns off, saccade to the remembered location of the cue. (**b**) Sequence of events in the ODR with distractor task. After the cue appearance and delay period, a distractor stimulus, which needs to be ignored, appears at a location diametric to the cue. The monkey is still required to saccade to the remembered location of the cue. (**c**) Possible locations of the stimulus presentation on the screen. (**d**) Performance in the ODR task, during neural recording sessions. Proportion of correct trials is plotted for each monkey, excluding trials that were aborted before the end of the delay period due to breaks in fixation. Young and adult sessions are represented in blue and red, respectively (*n*=134 sessions for young, 178 for adult). (**e**) Performance for the ODR with distractor task (*n*=32 sessions for young, 54 for adult).

We evaluated working memory performance with variants of the oculomotor delayed response (ODR) task (Fig. 1a–c). The basic task (Fig. 1a) required monkeys to observe a visual cue that could appear at one of eight locations (Fig. 1c) and, after a delay period, to make an eye movement to the location of the remembered visual stimulus (Fig. 1a). Recordings commenced once each monkey reached asymptotic performance and no further consistent improvement was observed during experiments (Supplementary Fig. 1). Young monkeys achieved an average of 86% correct responses (Fig. 1d) in 19,560 trials that had not been aborted by the end of the delay period because of a break in fixation^25^. The monkeys additionally made a number of errors (premature eye movements or breaks in fixation), which could occur at any task epoch before the end of the delay period (Supplementary Fig. 2A). These responses indicate a reliable performance level compared with reports of adult monkeys in the literature^19^,^26^. However, we used a relatively short (1.5 s) delay period compared with the standard of 3 s or longer in previous studies to ensure that the young animals would be able to perform the task. When the same four monkeys reached adulthood, asymptotic performance in the same task had increased to an average of 97% correct, in 20,980 trials. This represented a highly significant difference (two-way analysis of variance (ANOVA) with factors young/adult stage, and individual monkey; *F*_1,304_=189.7 for main effect of stage, *P*=7.2 × 10^−34^).

Two young and two adult monkeys (with one animal being tested in both stages) were also tested with a variant of the ODR task involving the presentation of a distractor (ODR+d task, Fig. 1b). In this task, the monkeys must still make an eye movement towards the remembered stimulus, but a distracting stimulus appears in the middle of the delay period. In general, performance was slightly lower in the ODR+d than the ODR task (Fig. 1e). Again, performance in completed trials improved significantly over time in the animal tested at both stages (two-tailed *t* test, *t*_49_=4.38, *P*=6.2 × 10^−5^). Young monkeys made more premature errors as well, particularly in the second delay period (Supplementary Fig. 2B).

### Neural activity during working memory

**Figure 2:**
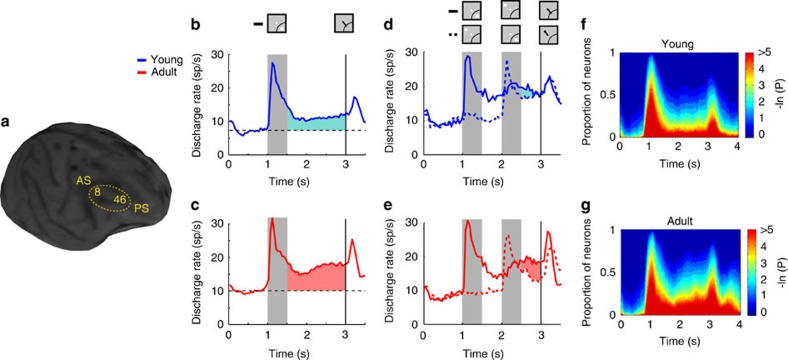
Neural activity in working memory tasks. (**a**) Magnetic resonance imaging (MRI) image of one young monkey with areas of recording indicated. (**b**) Average, population peri-stimulus time histogram for neurons that responded to the visual stimulus and were recorded during the ODR task in the young stage (*n*=309). Responses are shown for either a stimulus in the neuron's receptive field (solid line) or diametric to it (dotted line). Gray bar represents interval of stimulus presentation; vertical line represents the time of fixation offset. Insets show schematically the stimulus location and direction of eye movement relative to the receptive field (arc), which varied for each neuron. (**c**) As in *B*, for the adult stage (*n*=324 neurons). (**d**,**e**) Average population peri-stimulus time histograms (PSTH) for the ODR with distractor task (*n*=94 for the young, *n*=122 for the adult stage). (**f**,**g**) Percentage of neurons with significant difference in firing rate compared with baseline fixation (colour scale represents probability of a paired *t* test), at different time points of the ODR task, for the young (F) and adult (G) groups.

Neurophysiological recordings were obtained from the four monkeys during performance of the working memory tasks in each of the two developmental stages. A total of 607 neurons were recorded from areas 8a and 46 of the dorsolateral prefrontal cortex (Fig. 2a) in the young stage (*n*=33, 133, 158 and 283 neurons for the four monkeys, respectively). An additional 830 neurons were recorded in the adult stage from the same monkeys (*n*=133, 41, 238 and 418, respectively). To make an unbiased comparison of activity related to working memory, we selected neurons that responded to visual stimuli and then examined their activity during the delay period of the task, so that the presence of delay-period activity was not a criterion for selection. A total of 309 neurons in the young stage and 324 neurons in adult stage responded significantly to at least one visual stimulus during the ODR task compared with baseline activity (evaluated with a paired *t* test, at the *P*<0.05 level), and were selected for further analysis. Results did not differ appreciably if we relied on a broader or narrower selection criterion (discussed below). Approximately half of these neurons also exhibited delay period activity that was selective for the location of the stimulus (young 46% and adult, 49%). An additional 248 neurons in the young period and 258 neurons in the adult stage were tested with the ODR with distractor task, in two monkeys. Of those, 94 neurons responded to visual stimuli in the young stage and 122 in the adult stage and were further analysed.

An increase in delay period activity was evident in the adult stage relative to the young stage. We compared activity in the two stages in three ways: via the absolute firing rate during the delay period; via the firing rate activity expressed as a percentage of the cue response; and via the difference in delay period firing rate relative to that in the baseline fixation interval. The mean firing rate across all neurons during the best delay period was 11.3 spikes per s in the young stage and 17.0 spikes per s in the adult, a highly significant difference (two-tailed *t*-test, *t*_631_=5.15, *P*=3.4 × 10^−7^). This represented 56% of the response to the stimulus in the young period and 71% of the stimulus response in the adult, which also represented a significant difference (*t*-test, *t*_631_=5.95, *P*=4.4 × 10^−9^). The mean delay period rate relative to the baseline was 3.8 spikes per s in the young stage (shaded blue area in Fig. 2b) and a 6.9 spikes per s in the adult stage (shaded red area in Fig. 2c). This difference between stages was also significant (*t*-test, *t*_631_=4.39, *P*=1.3 × 10^−5^). As a result of this general increase in delay period activity, higher percentages of neurons exhibited significantly elevated activity at each time point of the delay period over the baseline period (Fig. 2f,g). Importantly, differences in activity during the cue presentation period were much smaller. The mean evoked response during the cue period relative to the baseline was 11.9 spikes per s for the young and 12.6 spikes per s during the adult stage, a non-significant difference (*t*-test, *t*_631_=0.98, *P*=0.33).

Increases in delay period activity in the adult stage were consistent within individual animals and within prefrontal subdivisions. For two monkeys we had sufficient neurons recorded in both the young and the adult stage for a comparison between stages (Supplementary Fig. 3). Absolute firing rate was significantly higher in the adult stage compared with the young stage in each monkey (one-tailed *t*-test, *t*_159_*=*3.00, *P*=1.6 × 10^−3^ and *t*_349_*=*4.14, *P*=2.2 × 10^−5^, respectively). Delay period activity expressed as a percentage of cue period responses was similarly significantly higher in each monkey (*t*-test, *t*_159_*=*2.28, *P*=0.012 and *t*_349_*=*5.04, *P*=3.7 × 10^−7^, respectively). Delay period activity above the baseline was also higher in the adult versus the young stage, though the difference reached statistical significance in only one animal (*t*-test, *t*_159_*=*1.64, *P*=0.052 and *t*_349**_=3.63, *P*=1.6 × 10^−4^, respectively). Differences in activity were also evident in the adult stage within different prefrontal subdivisions (Supplementary Fig. 4). When we examined separately areas 8a and 46 we saw an increase in adult delay period activity (*t*-test, *t*_314_=2.69, *P*=3.8 × 10^−3^ and *t*_303_=4.85, *P*=9.9 × 10^−7^ for the two areas, respectively).

Our analysis so far was based on neurons that responded during at least one stimulus of the ODR task. To ensure that differences between stages were not due to this selection criterion, we broadened our analysis and compared activity of all (607 and 830) neurons recorded in the two stages (Supplementary Fig. 5). Significantly higher delay period activity in the adult stage was evident in the absolute level of delay period activity (one-tailed *t*-test, *t*_1435_=4.33, *P*=8.0 × 10^−6^), in the percentage of cue activity that delay period activity represented (*t*-test, *t*_1435_=5.34, *P*=5.5 × 10^−8^), and in the difference from baseline (*t*-test, *t*_1435_=2.52, *P*=5.9 × 10^−3^). We used this population of neurons to also more fully characterize differences between stages across all task epochs. We thus found no significant difference in the inter-trial interval (mean discharge rate in young stage: 9.9 spikes per s, in adult: 9.5 spikes per s; two-tailed *t*-test, *t*_1435_=0.585, *P*=0.56). An increase in firing rate emerged in the fixation period (young 7.6, adult 9.6 spikes per s; two-tailed *t*-test, *t*_1435_=3.59, *P*=3.4 × 10^−4^). No further increase in excitability in the adult stage, relative to the fixation period, was evident during the presentation of the stimulus (mean rate–fixation rate in the young stage 7.6, in the adult 6.6 spikes per s; two-tailed *t*-test, *t*_1435_=1.89, *P*=0.06). Activity after the saccade termination and delivery of reward also did not differ between stages (young 4.3, adult 4.2 spikes per s; two-tailed *t*-test, *t*_1435_=0.233, *P*=0.82).

Conversely, we narrowed our selection criterion and examined only those neurons with delay period activity that was significantly selective for the location of the stimulus (one-way ANOVA, *P*<0.05). These neurons could be most informative about the location of the stimulus held in memory. A total of 46% (143/309) of task-selective neurons were selective for the stimulus location during the delay period in the young stage and 49% in the adult (158/324). A higher firing rate was evident for the adult group too (one-tailed *t*-test, *t*_299**_=2.06, *P*=0.02).

We proceeded to analyse delay period activity in the ODR+d task, which required maintaining in memory the location of an initial cue stimulus and ignoring a subsequent distractor. Delay period activity followed the initial stimulus that needed to be remembered when that stimulus appeared in the receptive field (Fig. 2d,e, solid lines), but was also present after the distractor when it appeared in the receptive field, following presentations of the cue stimulus out of the receptive field (Fig. 2d,e, dotted lines). During the crucial second delay interval, activity representing the initial stimulus was higher than activity representing the distractor (shaded area in Fig. 2d,e). This difference, which represents a measure of the ability to resist the effect of the distractor^27^, was significantly larger in the adult stage compared with the young stage (two-tailed *t*-test, *t*_191_=2.04, *P*=0.04).

### Stimulus discrimination

**Figure 3:**
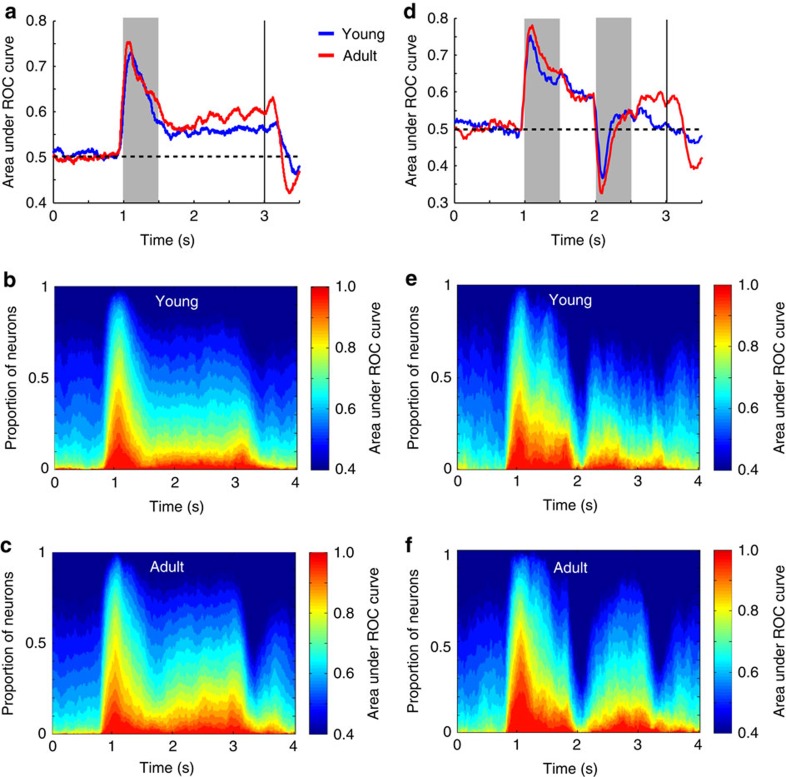
ROC analysis in working memory tasks. (**a**) Area under ROC curve in successive 250 ms windows is plotted as a function of time during the ODR task, for the young (average of *n*=309 neurons) and adult stage (*n*=324). (**b**) Percentage of neurons in the young age reaching different levels of ROC values at each time point of the ODR task. (**c**) Percentage of neurons in the adult age reaching different levels of ROC values at each time point of the ODR task. (**d**) Area under ROC curve in successive 250 ms windows is plotted as a function of time during the ODR+d task for the young (*n*=94) and adult stage (*n*=122). (**e**,**f**) As in **b**,**c**, for the ODR+d task.

Improved performance in the task may also be related to the ability to better discriminate between stimulus locations, which refers to the spatial selectivity of the neurons rather than their absolute level of activation. To test this possibility, we evaluated discriminability between the best location and its opposite, using a receiver operating characteristic (ROC) analysis. Mean ROC values computed during the stimulus presentation period of the ODR task (Fig. 3a) were indistinguishable between the young and adult stages (two-tailed *t*-test, *t*_631_=0.36, *P*=0.72). In contrast, mean ROC values for the delay period were significantly higher in the adult stage (*t*-test, *t*_631_=2.0 *P*=0.046). This was the result of larger percentages of neurons in the adult reaching higher ROC values at each time point of the delay period (Fig. 3b,c). In the ODR+d task (Fig. 3d–f), the ability of neurons to discriminate between the best location and its opposite in the delay period, after the appearance of a distractor, was also higher in the adult compared with the young stage (*t*-test, *t*_214_=2.52, *P*=0.01).

**Figure 4:**
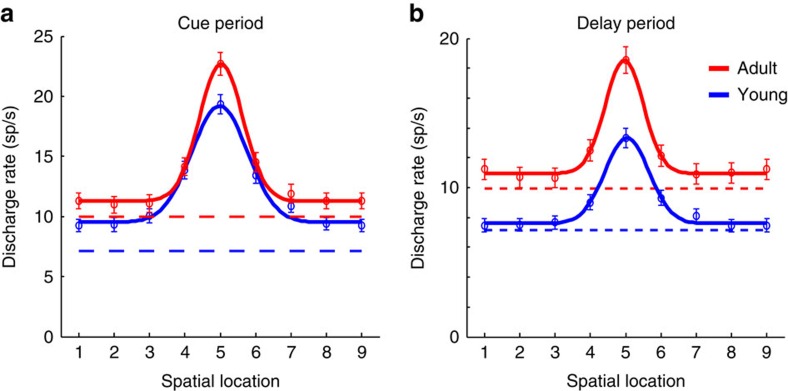
Tuning curve. (**a**) Average activity (and s.e.m.) during the cue period of the ODR task in neurons recorded during the young (*n*=309) and adult stage (*n*=324). Locations have been rotated, so that the best location of each neuron is represented in location 5. Location 9 is the same as location 1. Solid lines represent best Gaussian fit of the population average. (**b**) Average activity (and s.e.m.) during the delay period of the ODR task in the same sample of neurons.

The adult stage was associated with an increase in the absolute level of activity for all stimulus locations, both during the cue presentation and delay period (Fig. 4). A two-way ANOVA of firing rate, with factors cue location and young/adult stage revealed a significant effect of stage (*F*_1,5679_=24.75, *P*=6.7 × 10^−7^ for the cue period, *F*_1,5679_=147.63, *P*=1.5 × 10^−33^ for the delay period). This increase was greatest for the best location, and the relative difference more pronounced in the delay than the cue period (Fig. 4a,b). A subtle difference between stages involved the width of tuning. The s.d. of the population tuning curves was 35.4° in the young and 27.6° in the adult for the cue period, a significant difference (permutation test, *P*<0.005), and 28.1° in the young versus 24.2° in the adult for the delay period, though the latter contrast did not reach statistical significance (permutation test, *P*>0.1).

**Figure 5:**
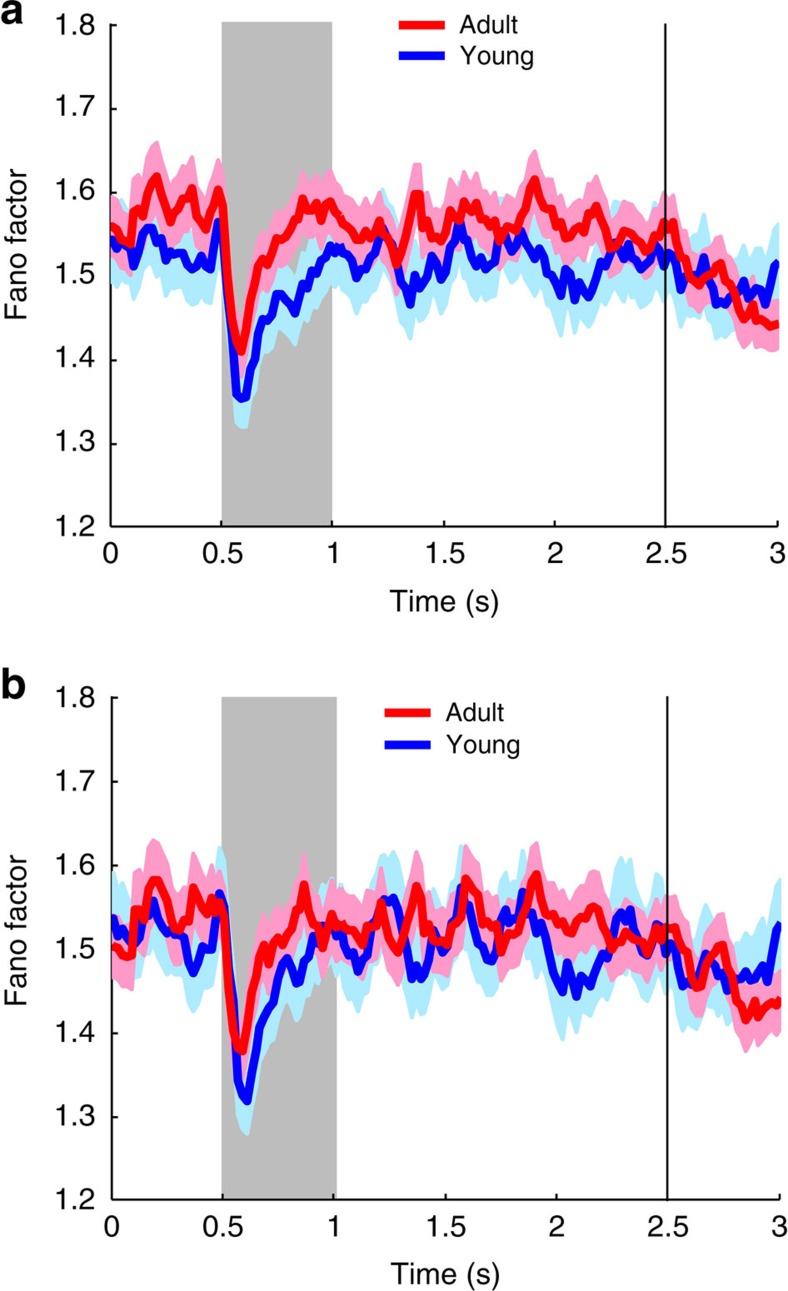
Variability of neuronal responses. (**a**) Fano factor of spike counts is plotted as a function of time in successive time points (partially overlapping 100 ms windows), for the young (*n*=309 neurons) and adult (*n*=324) stage. (**b**) Fano factor is plotted as in **a**, but based on subsets of neurons matched for firing rate (*n*=231 for the young, 231 for the adult).

Differences in discriminability depend not only on mean firing rate but also on the variability of neuronal responses; reduced variability may improve the information represented in cortical circuits^28^,^29^. We therefore sought to test whether the adult prefrontal cortex is characterized by reduced variability by examining the Fano factor of spike counts. This was not the case. Overall, Fano factors were slightly higher in the adult than in the young prefrontal cortex (Fig. 5a). Since firing rate was higher overall, we also performed a comparison of Fano factor values in samples of neurons in the young and adult prefrontal cortex after matching neurons for firing rate (Fig. 5b). No significant difference in Fano factor was present for either the fixation, cue, or delay period (*t*-test, *P*>0.05 in every case).

Stimulus and task information may be represented in neuronal activity even in time periods when no significant changes in mean firing rate are evident, based on dynamic patters of population responses^30^. To investigate potential substrates of maturation that improve the ability to represent information but may not be evident in the averaged firing rate across the population of neurons, we evaluated the accuracy of a classifier^31^,^32^ in determining the correct stimulus location. The classifier was tested based on the simultaneous responses of 500 neurons, in the young and adult stage (Supplementary Fig. 6), sampled randomly from all available neurons, whether they exhibited significantly elevated responses to the visual stimuli or not. Peak classification accuracy during the cue period was indistinguishable between the young and adult stages. However, classification was consistently higher during the delay period of the task, and this difference was statistically significant (permutation test, *P*<0.005, corrected for multiple comparisons).

### Relationship between performance and activity

Previous studies have shown that reduced levels of prefrontal activity during the delay period of the ODR task are more likely to result in errors^19^,^25^. On the other hand, responses in correct trials do not indicate a monotonic relationship between delay period activity and overall performance across sessions. For example, aged monkeys are able to perform the ODR task at the same levels of adult monkeys, yet delay period activity is markedly lower in aged than adult animals^33^. To untangle the effects of performance improvement in the task on firing rate comparisons, it was essential to examine more carefully the relationship between performance and delay period activity in the task.

**Figure 6:**
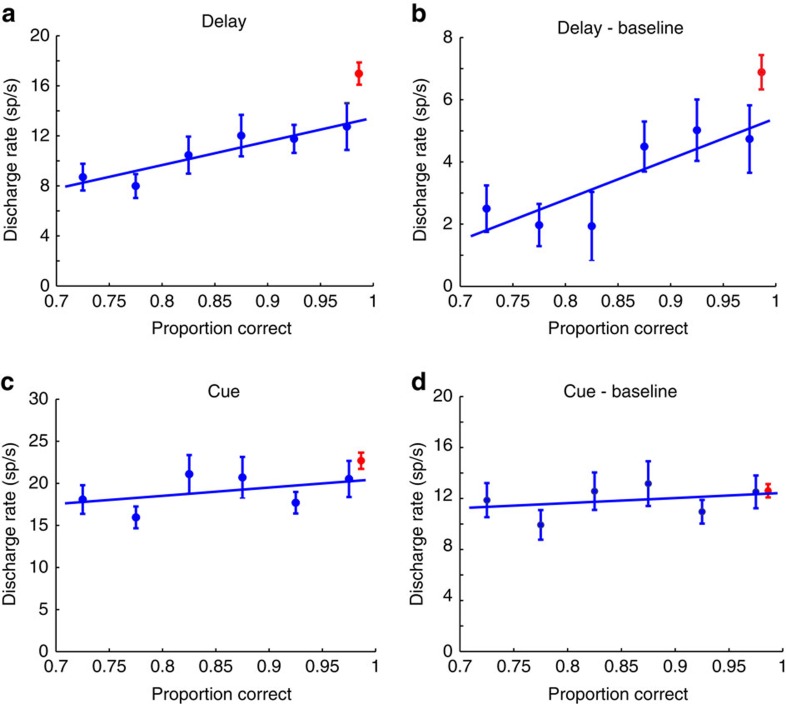
Neuronal activity as a function of performance. (**a**) Average delay period activity (and s.e.m.) for the best stimulus location is plotted for groups of neurons recorded from young monkeys in sessions that fall within each bin of performance (blue points, *n*=29, 41, 43, 40, 58, 79 for the six data points, respectively). Regression line is superimposed over individual points. Red point represents average delay period activity of all neurons recorded in the adult PFC, plotted at the average adult performance. (**b**) As in **a**, but for delay period activity minus the baseline (fixation period) activity, for the same groups of neurons. (**c**) As in **a**, but for cue period activity. (**d**) As in **a** but for cue period activity minus the baseline.

The relatively low and considerably variable levels of performance in the young stage allowed us to determine the relationship between performance and prefrontal activity. That is, we examined how discharge rate varied as a function of performance across sessions (Fig. 6a,b). Six groups of sessions were identified, spanning a range in performance of 25%, which was higher than the overall improvement we saw between the young and adult stages. A systematic relationship between performance and delay period activity was present (regression analysis, *P*<0.01) as well as between performance and delay period activity after subtracting the baseline activity (*P*<0.05). The delay period activity of neurons recorded in the adult stage and the respective performance produced a point that fell above the regression line (red point in Fig. 6a), predicted on the basis of performance levels alone.

This stage-related improvement in activity, beyond the level predicted by performance was specific for the delay period. Performance had no significant predictive ability on the activity evoked by the cue (regression analysis *P*>0.3). A trend was still present towards higher cue period activity for sessions of higher performance, and adult cue responses also fell above the regression line (Fig. 6c), however, these two effects were almost entirely accounted for by a shift in baseline activity already present in the fixation period. After subtracting the baseline activity, performance was entirely non-predictive of evoked cue responses (regression analysis, *P*>0.5, *R*^2^=0.096). Adult stage cue responses above the baseline were also in line with the level of performance achieved in that stage (Fig. 6d).

These results suggest that delay period activity but not cue activity increased in the adult stage beyond what would be predicted by the increase in performance alone. We explicitly tested this hypothesis by repeating our comparison of firing rate between stages across all neurons (not binned as in Fig. 6) including performance during the session as a covariate in an analysis of covariance. A significant effect of stage was present for delay period activity (analysis of covariance, *F*_1,630_=12.14, p=5.3 × 10^−4^) and for delay period activity above the baseline (*F*_1,630_=4.76, *P*=0.029). On the other hand, a less robust effect of stage was observed for cue activity (*F*_1,630_=4.27, *P*=0.039), and no significant effect was present for cue activity after subtracting the baseline (*F*_1,630_=0.11, *P*=0.74).

We reached the same conclusion when we limited our comparison to a range of performance over which young and adult behaviour overlapped (Supplementary Fig. 7). By selecting the best sessions from the young and worst sessions from the adult stage we were able to identify recordings from 98 young and 133 adult neurons, in samples with no significant difference in performance (mean performance 96% in the young; 97% in the adult). This analysis, too, confirmed a higher level of activity in the delay period of the adult prefrontal cortex (PFC) compared with the young stage, a difference that was statistically significant (two-tail *t*-test, *t*_229_=2.69, *P*=7.7 × 10^−3^). In contrast, the difference in cue period activity failed to reach significance (*t*-test, *t*_229_=1.74, *P*=0.08).

### Stability of delay period activity changes

The monkeys tested during the adult stage had more cumulative exposure to the task than in the young age, as an inevitable consequence of our longitudinal experimental design. To evaluate the effect of exposure to the task, and the sampling of neurophysiological activity at two time points years apart, we conducted a third stage of recordings in two monkeys. Recordings of this, second adult, stage began at an age of 8.2 and 8.3 years respectively, which represented an interval of 2.0 and 2.7 years after the onset of the first adult stage (which in turn occurred 2.1 and 1.6 years after the young stage, in these animals). The monkeys had continued to be exposed to the ODR task between the first and second adult stage of recordings, and recordings were obtained from other cortical areas for the purposes of other experiments^34^. A total of 479 neurons were recorded in a third stage of recordings (310 and 169 neurons from the two respectively). Of those, 130 neurons responded during at least one task period, and were recorded in sessions matched for behavioural performance with the performance of the first adult stage (75 and 55 neurons from the monkeys, respectively). A total of 656 neurons were recorded from the same animals in the initial adult stage, 274 of which responded to stimuli and were recorded in sessions matched for behavioural performance.

**Figure 7:**
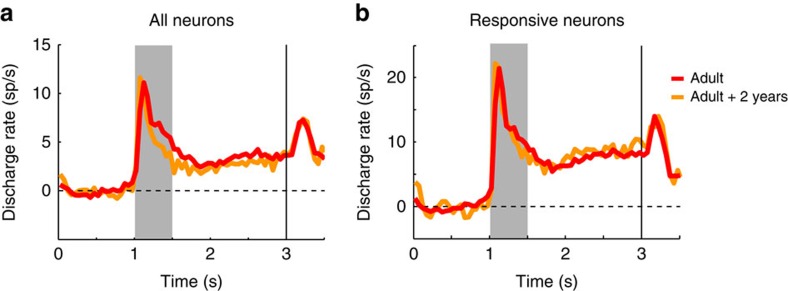
Neural activity from second stage of adult recordings. (**a**). Average population peri-stimulus time histograms (PSTH) in the ODR task from two monkeys in the initial adult stage (*n*=656 neurons) and a second time point of the adult stage, obtained ∼2 years after the monkeys reached adulthood. Activity from all available neurons is averaged together (*n*=479). (**b**) Average population PSTH of neurons activated by at least one stimulus and recorded in sessions of the ODR task matched for behavioural performance, in the initial adult (*n*=274 neurons), and second adult stage (*n*=130 neurons).

Discharge rate in delay period minus the baseline (the best predictor of performance and developmental stage, identified above) was then compared between the two adult stages. Among all available neurons (Fig. 7a), mean rate in the initial adult stage was 3.3 spikes per s compared with 2.7 spikes per s in the later adult stage, which represented no significant difference (two-tailed *t*-test, *t*_1133_=1.28, *P*=0.20). Restricting the analysis to neurons responding to stimuli and recorded in sessions matched for behavioural performance yielded very similar rates between the two adult stages (Fig. 7b). Mean rates were 7.5 spikes per s in the initial and 8.0 spikes per s in the later, which were not significantly different from each other (*t*-test, *t*_402_=0.46, *P*=0.65). The results confirm the reliability of delay period activity measures recorded in experiments years apart, and indicate that delay period relative to baseline is a robust indicator of developmental stage.

## Discussion

Our findings establish non-human primates as a model of cognitive development that mirrors the progression of working memory ability observed in humans during adolescence^1^,^2^,^3^,^4^. Similar to previous studies of development around the time of puberty^35^, we used a longitudinal study designed to track monkeys at different time points, and we additionally recorded neuronal activity from the prefrontal cortex. This allowed us to detect subtle changes in performance and neuronal activity within subjects. Inevitably, this also meant that adult animals had higher cumulative exposure to the task. However, exposure alone did not explain differences in neural activity we observed between stages. Little difference was present in evoked activity in adult animals tested at a time point approximately equal to the interval between the young and adult stages, after the monkeys continued to be exposed to the task. Performance of the task at a higher level in the adult stage did not fully account for our findings, either. When we analysed neural data from the young stage obtained from sessions in which the monkeys achieved performance equal to that seen in (a subset of) adult sessions, delay period activity was still lower in the young stage.

Our findings identify aspects of prefrontal neural activity that differed between developmental stages. The adult stage was characterized by greater delay period activity for actively remembered stimuli and reduced delay period activity for distractors. Evoked firing rate in the delay period relative to the baseline was the best indicator of developmental stage. On the other hand, the firing rate evoked during stimulus presentation and the discriminability of stimulus location were similar across stages, suggesting that the representation of visual stimuli was mature at the time of puberty. Variability of spike rates (quantified by the Fano factor) exhibited no difference between stages, and tuning of neuronal responses, only subtle differences. These results identify key neural correlates of the developmental processes that correlate with enhanced working memory ability in adolescence.

Development of working memory ability in monkeys begins in infancy. Monkeys younger than 1 year of age are able to perform delayed response and delayed alternation tasks at high levels^36^,^37^. Three-year-old monkeys can perform more complex working memory tasks, such as the delayed match to sample task, at modest levels^38^. Our results indicated that monkeys at a developmental stage after the onset of puberty can perform the ODR and ODR with distractor tasks robustly, at least with a relatively short 1.5 s delay period. However, further improvements in performance were evident for the same animals between puberty and adulthood. We selected this short delay period interval at the onset of experiments (compared with 3 s or longer delay periods used in studies of adult animals^19^) to ensure that training would be complete before the monkeys reach adulthood, and that collection of sufficient numbers of trials would be possible from neurophysiological recordings. It remains to be seen if performance in young animals is disproportionately affected by longer delay periods, or more complex working memory tasks.

The neurophysiological responses we observed during working memory performance in adulthood were generally in agreement with findings from prior studies of adult monkeys^19^ and allowed comparison with responses of the same animals around the time of puberty. The greatest difference in adulthood involved more robust delay period activity. This corresponded to higher absolute discharge levels, a greater increase from the baseline firing rate of neurons, and maintenance of a higher percentage of the activation elicited by the cue into the delay period. Furthermore, discriminability between the spatial locations of the stimuli, estimated with ROC analysis and classifier performance was enhanced in adulthood, specifically for the delay period. We should note that activity elicited by the ODR task reflects several processes, including retrospective memory (the representation of the remembered location of the cue), prospective memory (the representation of the upcoming target location) and preparation for the saccade^39^. Our results cannot resolve how developmental differences affected each; more complex memory paradigms will be required for addressing this question. In humans, improvement in visuo-spatial performance and other cognitive functions is characterized by greater lateralization of activity that occurs after the time of puberty and tends to be sex-specific^40^,^41^. Differences may be greater in the right hemisphere (which we recorded from) than in the left, and in male monkeys (as our subjects) than female. Examples of lateralization in neural activity have not been reported for neurophysiological activity in monkeys and it is on future studies to address whether such differences are present.

It was notable that these changes were specific for the delay period of the working memory task. No difference in evoked firing rate (above the baseline) was observed between stages for the cue period, and ROC analysis revealed no advantage in cue discrimination in the adult stage. These results suggest that stimulus representation in the prefrontal cortex is essentially mature at the time of puberty. Our experiments additionally revealed a relative decrease in the activity evoked by a distractor in the ODR with distractor task. This finding is consistent with a more robust representation in memory of behaviourally relevant stimuli in adulthood. Although improved representation of information can be achieved through changes in variability in individual neural responses^28^,^29^, we found no evidence that this mechanism plays a major role during the transition to the adult stage. Fano factor values were no lower during adulthood than adolescence (Fig. 5). Instead, firing rate during the delay period represented the main substrate of developmental changes.

The role of persistent activity as the neural basis of prefrontal activity has come under question, in recent years^42^. Alternative mechanisms, including synaptic changes in neuronal connections^43^, dynamic patterns of activation of specific neurons in the absence of overall increases in activity during the delay period^30^, and bursts of rhythmic responses^44^ have been proposed as the critical factors mediating working memory, instead. While we cannot rule out the role of these mechanisms in working memory, our present results indicate that delay period activity predicts behavioural performance in adolescence and undergoes developmental changes, at least for spatial working memory, for which persistent discharges have been shown to precisely predict behavioural outputs in the ODR task^45^.

Delay period activity in the prefrontal cortex represents stimulus attributes^46^ and parameters of working-memory related behaviour^45^. In light of the behavioural improvement in adulthood, the finding of increased prefrontal activity we report here might be seen as an inevitable consequence of this higher level of performance. Analysis of the relationship between activity and performance revealed a more complex picture. Increased prefrontal activity was indeed observed in sessions with higher overall performance within a developmental stage. However, we found that the activity increase between stages was beyond what would be expected by the observed improvement in performance alone. It appears that post-pubertal development resulted in increased prefrontal period activity that could fully account for the improved performance as well as an excess increase in activity not directly predicted by behaviour. An analogous difference in prefrontal neuronal responses has been observed between adult and aged monkeys, with adult animals exhibiting higher levels of activity specifically in the delay period of the ODR task, despite similar levels of performance in the adult and aged animals^33^. The presence of increased delay period activity observed in this study and in ours may represent a form of cognitive reserve on which more complex working memory tasks depend, or may have contributed to success in task performance that was beyond our criterion for accuracy. Collectively, our results identify increased delay period activity as a critical substrate of developmental changes between the time of puberty and adulthood.

## Methods

### Developmental profiles

The subjects used in this study were male macaque monkeys (*Macaca mulatta*). Animal use procedures reported here were reviewed and approved by the Wake Forest University Institutional Animal Care and Use Committee, in accordance with the U.S. Public Health Service Policy, as informed by the National Research Council's Guide for the care and use of laboratory animals. The study compares measures of working memory performance and neural correlates of working memory in two stages, during puberty and adulthood. To obtain these, it was necessary to track developmental measures and to ascertain the onset of puberty. The monkey life span is ∼25 years in the wild, and up to 40 in captivity, indicating a rate of aging of ∼3 times that of humans^47^,^48^. The age of puberty and full sexual maturity can vary considerably between individuals, however. It was necessary, therefore, to obtain developmental measures around the time of the experiments, which we did in a quarterly basis. The onset of puberty was determined based on morphometric measures including body weight, crown-to-rump length, chest circumference, ulna and femur length, and testicular volume (determined with a Prader Orchidometer, ESP Limited, Rustington, UK). We also checked for visible eruption of canines and bone maturation based on X-rays of the upper and lower extremities. Blood samples were used to estimate the serum concentration of testosterone and dihydrotestosterone through extraction and enzyme immunoassay (performed at the Assay Services Unit of the Wisconsin National Primate Research Center).

Body mass, bone length and testes size measurements during the young stage were all consistent with individuals in a growth trajectory, as we have documented elsewhere^49^. Canines had not erupted in 3/4 monkeys, and epiphyseal plates of extremities were open in 4/4 subjects, which are also signs of continued growth.

### Behavioural tasks

All monkeys were initially trained to perform the ODR task (Fig. 1a). This task required them to remember the spatial location of a 1° white cue stimulus presented on a screen for 0.5 s. The cue appeared pseudo-randomly at one of eight possible locations arranged on a circle of 10° eccentricity. The presentation of the cue was followed by a 1.5 s delay period, over which only the fixation point was visible on the screen. At the end of the delay period, the fixation point was extinguished and the monkey was required to make an eye movement to the remembered location of the cue within 0.6 s to receive a liquid reward. Correct responses were considered those in which the saccadic end point deviated no more than 5–6° from the centre of the stimulus (3–4° from the edge of the stimulus), and the monkey held fixation within this window for 0.1 s. Trials in which eye position failed to be maintained/at any point before the offset of the fixation point (monitored with an infrared eye tracking system: ISCAN, RK-716; ISCAN, Burlington, MA) were aborted immediately and the trial resulted in no reward. The visual stimuli were presented and the online control of behaviour was performed by in-house software^50^ developed in the MATLAB environment (Mathworks, Natick, MA).

In addition to the ODR task, two of the four young monkeys and two of the adult ones (three in total) were trained to perform the ODR with distractor task^51^ (ODR+d, Fig. 1b). This task also required the monkeys to remember the spatial location of a cue stimulus, but it additionally involved presentation of a second stimulus (distractor), which the monkeys needed to ignore. Delay periods intervened between the presentation of the stimuli, however, the total duration of the trial was 1.5 s, as in the standard ODR task. The cue could appear at any of eight locations used in the ODR task and the distractor was always diametric to it. Since the location of the distractor was predictable relative to the cue, it is possible that the distractor might have been used to plan the eye movement towards the rewarded location. Nonetheless, the ODR+d task allowed us to test the ability of the monkeys to correctly choose the initial over the second stimulus, and to determine the neuronal activity associated with it.

### Surgery and neurophysiology

The monkeys were initially naïve to behavioural training or task execution of any kind. They were first trained in the ODR and subsequently in ODR with distractor tasks during the young stage. The animals were additionally trained to perform anti-saccade tasks, data from which have presented elsewhere^49^. Once the young animals had reached asymptotic performance in their tasks, a craniotomy was performed and a 20-mm recording cylinder was implanted over the prefrontal cortex of the right hemisphere. Neurophysiological recordings could then be obtained from dorsolateral prefrontal areas 8a and 46. Positioning of the recording cylinder and localizations of electrode penetrations were aided with magnetic resonance imaging, processed with the BrainSight system (Rogue Research, Montreal, Canada). Epoxylite-coated Tungsten electrodes of 250 μm diameter and an impedance of 4 MΩ at 1 KHz (FHC Bowdoin, ME) were used in these experiments. The raw signal was amplified, band-pass filtered between 500 and 8 kHz, and stored for off-line analysis through a modular data acquisition system with 25 μs temporal resolution (APM system, FHC, Bowdoin, ME). Histological verification that would allow precise localization of areas was not available, but we distinguished between anterior recordings in the principal sulcus region (area 46) and posterior sites between the caudal end of the principal sulcus and the arcuate sulcus (area 8a) in each monkey, for some analyses. At the conclusion of these recordings, the animals were no longer tested or trained for a period of ∼1 year. After reaching adulthood, determined with the developmental indexes described above, the animals were again tested in the same tasks that they were originally trained. They were generally able to quickly re-master the tasks. A new stage of recordings was then performed from the same areas, using identical recording methods.

### Behavioural data analysis

We expressed correct performance in the ODR task and ODR with distractor task as the percentage of trials that resulted in correct responses. Some trials resulted in breaks in fixation, blinks or premature saccades, before offset of the fixation point. We therefore analysed performance in more detail, by expressing the percentage of correct trials after the offset of the fixation point, at the end of the delay period. Statistical comparisons were performed using a two-way ANOVA with factors young/adult stage, and individual subject. All analysis of behavioural (and neural) data were performed in the MATLAB environment.

### Neural data analysis

Recorded spike waveforms were sorted into separate units using an automated cluster analysis method based on the KlustaKwik algorithm^52^. Firing rate of units was then determined by averaging spikes in each task epoch. In the ODR task, we identified neurons with significant elevation of firing rate in the 500 ms presentation of the cue. Firing rate in this period was compared to the 1 s baseline fixation period, before the presentation of the cue, and neurons with significant difference in firing rate were identified (paired *t*-test, *P*<0.05). Neurons with significant responses during the stimulus presentation were used in further analysis presented here.

Population peri-stimulus time histograms were constructed averaging responses of multiple neurons. Statistical comparisons in the ODR task involved firing rates distributions recorded during the delay period of the task.

ROC analysis was performed, comparing the distribution of responses to the best location and the location diametric to it. The area under the ROC curve represents the probability that an ideal observer can discriminate between a stimulus appearing in the overall best and diametric location firing rate in each trial, based on the relative difference in firing rate between the two stimulus conditions^53^. The analysis was performed for spikes recorded during the entire cue period and delay period, and also in a time-resolved fashion, in sliding 100 bins, stepped every 10 ms.

Decoding accuracy was evaluated based on the accuracy of a correlational classifier to determine which stimulus was presented based on the responses of 500 neurons, which were treated as if they were recorded simultaneously^32^. This analysis was performed in a time-resolved fashion in sliding 500 ms bins, computed every 50 ms. The classifier computes correlation values across the vector of neuron responses for each stimulus condition, in essence discounting the absolute firing rate of individual neurons.

Some comparisons involved responses recorded in sessions equated for performance, doing so separately for each monkey. Sufficient data from two monkeys were available for these comparisons. We identified the set of highest performance sessions during the young stage and lowest performance sessions in the adult stage (mean performance for young 96%; for adult 97%), so that there was no significant difference in performance drawn from the two stages. We then proceeded to analyse neuronal responses drawn from these sessions, as described above.

### Data availability

All relevant data and codes are available from the authors upon request.

## Additional information

**How to cite this article:** Zhou, X. *et al.* Neural correlates of working memory development in adolescent primates. *Nat. Commun.* **7,** 13423 doi: 10.1038/ncomms13423 (2016).

**Publisher's note:** Springer Nature remains neutral with regard to jurisdictional claims in published maps and institutional affiliations.

## Supplementary Material

Supplementary Information Supplementary Figures 1-7
