# Supplementary material for: Neural correlates of working memory development in adolescent primates
Source: Nat Commun. 2016 Nov 9;7:13423. doi: 10.1038/ncomms13423 (PMC5105196; doi:10.1038/ncomms13423)
Supplement: Supplementary Information — Supplementary Figures 1-7 [file ncomms13423-s1.pdf]

SUPPLEMENTARY FIGURES

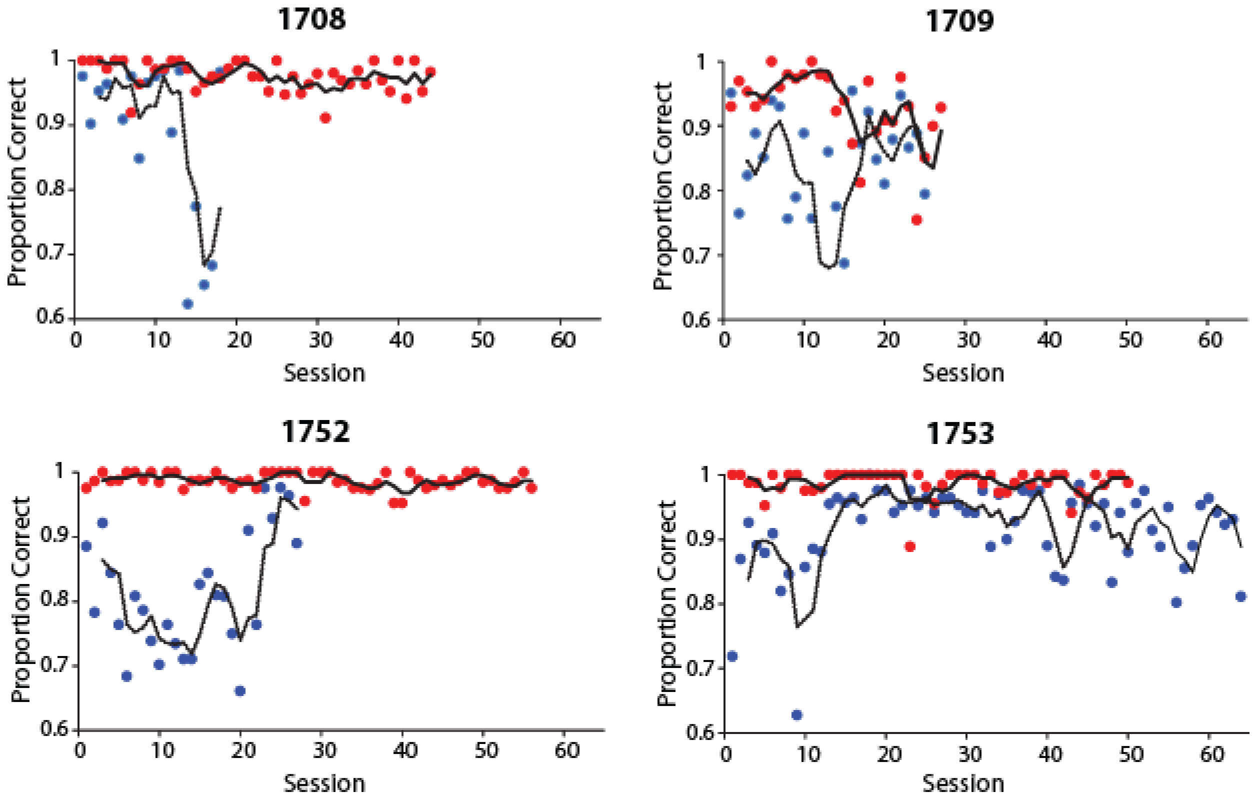

Supplementary Figure 1. Individual performance. Performance in the ODR task is plotted as a function of sequential session number, on successive recording days, for the young (blue dots) and adult (red dots) stage. Percentage of correct trials is plotted, for trials where the monkey did not break fixation until the end of the delay period, as in Fig. 1D. Lines represent 3-session running average through each data set.

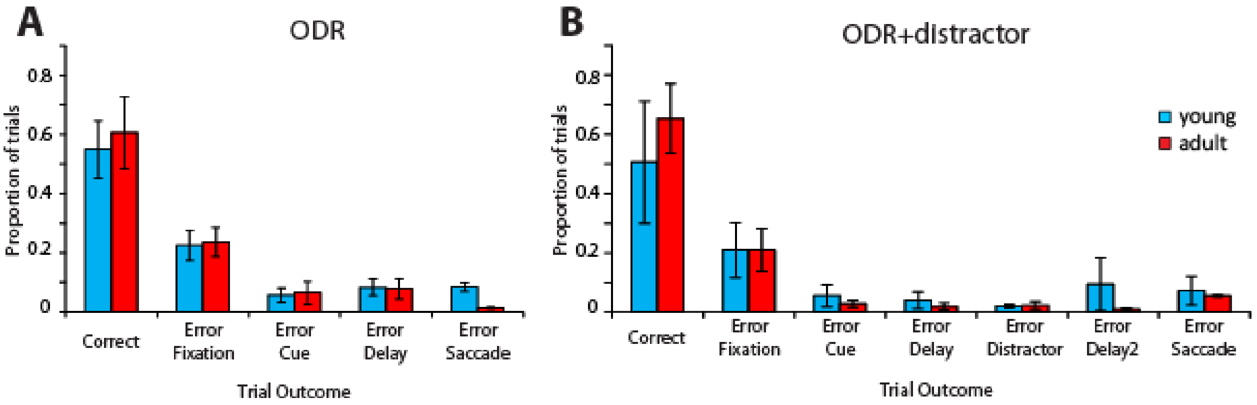

Supplementary Figure 2. Types of errors in working memory tasks. (A) Proportions of trials that ended in different types of behavioral outcomes are shown for the ODR task, in the young and adult stage. Histograms represent means of all sessions during which recordings were obtained (n=134 total sessions for young, 179 for adult). Error bars represent standard error of mean across performance of individual monkeys (n=4). Proportion of trials is depicted for errors that ended in correct outcomes, in errors during the fixation, cue, and delay periods (which were caused by premature eye movements or breaks in fixation), and during the saccade period (which were caused by saccades that failed to terminate in the target area). **(B)**. As in A, for the ODR+d task, for two monkeys, tested with this task.

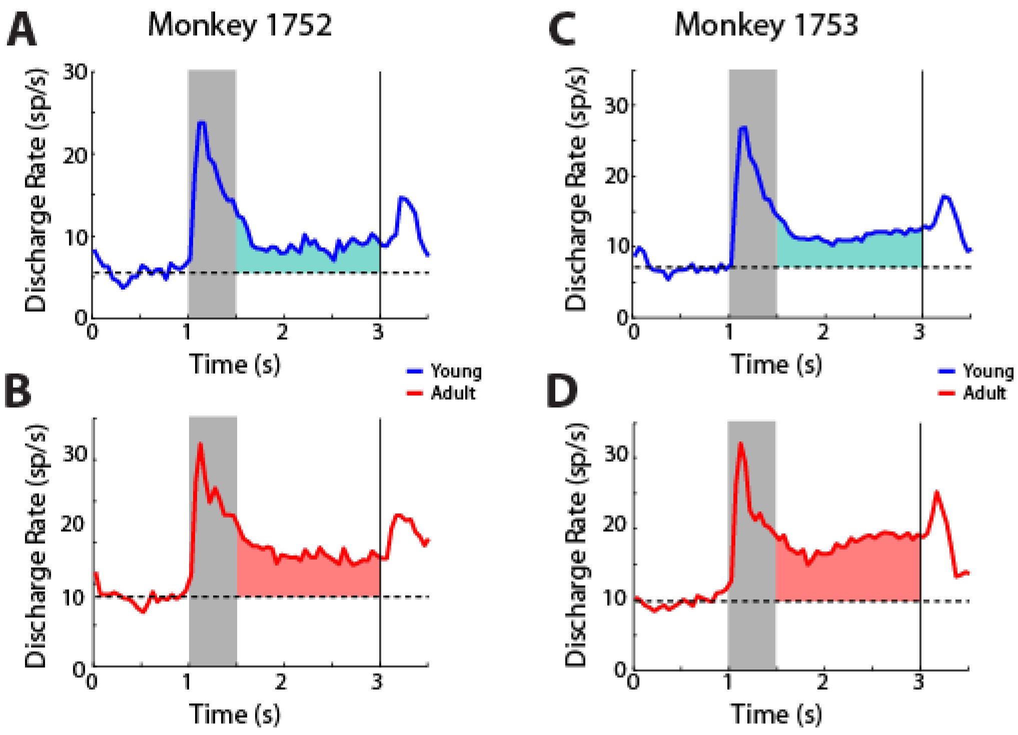

Supplementary Figure 3. Change in neural activity in the ODR task within monkeys. (A) Average population PSTH in sessions of the ODR task from one monkey in the young stage (n=88). Responses are shown for the best stimulus in the neuron's receptive field. Dotted line represents mean baseline, fixation rate. Gray bar represents time of stimulus presentation, vertical line the time of fixation target turning off. Insets above PSTH represent schematically the location of the stimulus relative to the receptive field (arc); this varied for each neuron. **(B)** As in *A*, for responses of the same monkey, in the adult stage (n=73 neurons). **(C-D)**. As in A-B, for responses of a second monkey in the young (n=150) and adult (n=201) stages.

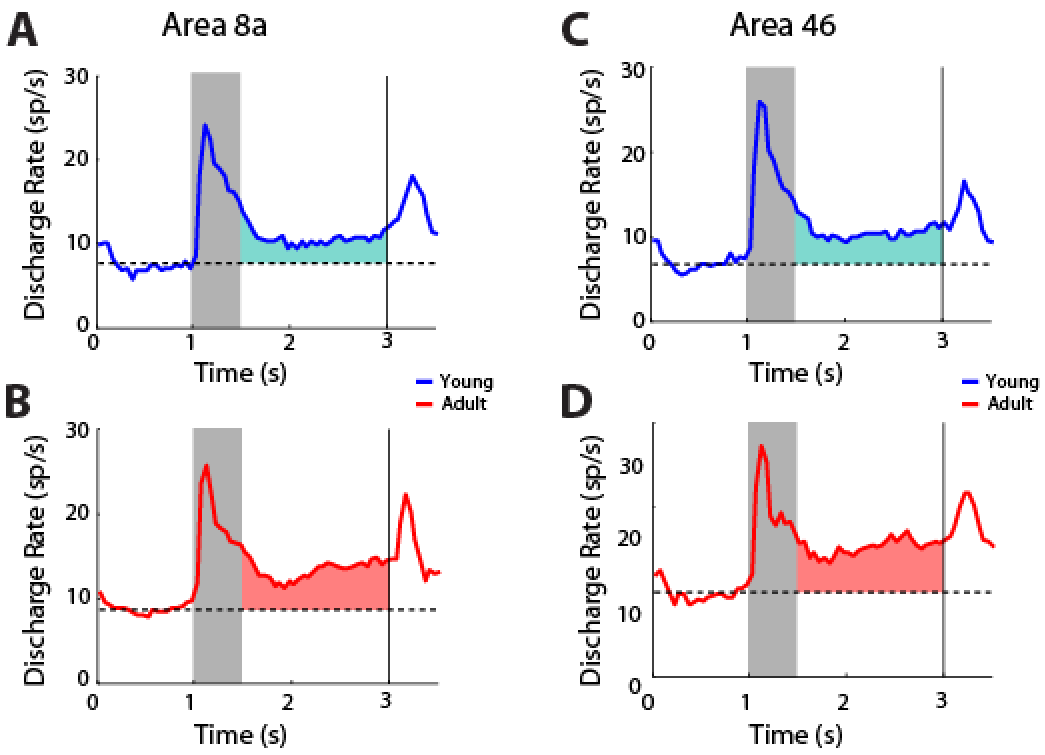

Supplementary Figure 4. Neural activity in area 8a and 46. (A) Average population PSTH from neurons recorded in area 8a in the young stage (n=117). Responses are shown for the best stimulus in each neuron's receptive field. Dotted line represents mean baseline, fixation rate. Gray bar represents time of stimulus presentation, vertical line the time of fixation target turning off. Insets above PSTH represent schematically the location of the stimulus relative to the receptive field (arc); this varied for each neuron. **(B)** As in *A*, for responses in area 8a, in the adult stage (n=199 neurons). **(C)** Population PSTH of neurons recorded in area 46 from the young stage (n=182). **(D)** Population PSTH of neurons recorded in area 46 in the adult stage (n=123).

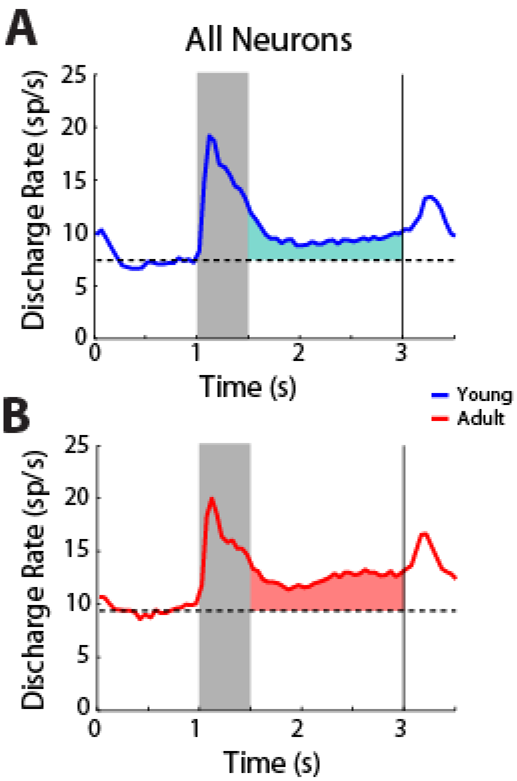

Supplementary Figure 5. Neural activity of all neurons recorded. (A) Average population PSTH from all neurons recorded in the experiments of the young stage (n=607), regardless of whether they responded at any epoch of the ODR task or not. **(B)**. Responses from all neurons recorded in the experiments of the adult stage (n=830).

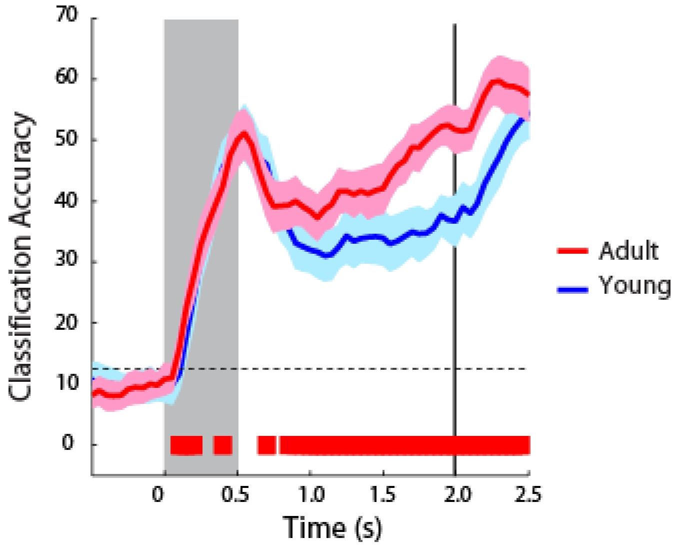

Supplementary Figure 6. Classification performance. Comparison of information about the location of a stimulus during the young (blue) and adult stage (red). Color shaded regions indicate 1 standard deviation of decoding accuracy, if different neurons were used. Gray bar represents time of cue presentation; vertical line represents time of fixation point turning off, which is followed by the saccade. Dotted horizontal line represents chance performance of decoding. Red bars at the bottom of the figure indicate times when the decoding accuracy of the two populations was significantly different from each other (permutation test, p<0.005, corrected for multiple comparisons).

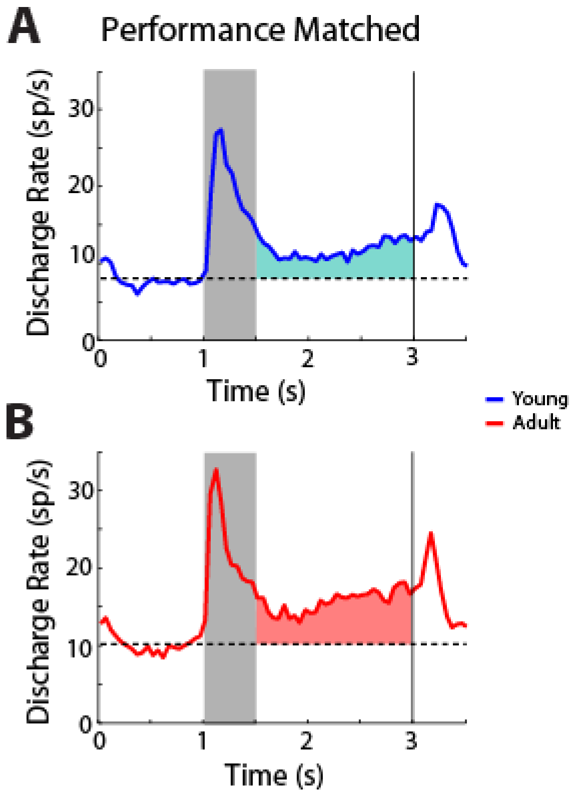

Supplementary Figure 7. Activity matched for behavioral performance. (A) Average population PSTH in sessions of the ODR task from two monkeys in the young stage (n=98 neurons), matched for behavioral performance for sessions in the adult stage. Responses are shown for the best stimulus in the neuron's receptive field. Dotted line represents mean baseline, fixation rate. Gray bar represents time of stimulus presentation, vertical line the time of fixation target turning off. Insets above PSTH represent schematically the location of the stimulus relative to the receptive field (arc); this varied for each neuron. **(B)** As in *A*, for responses of the same two monkeys, in the adult stage (n=133 neurons).
